# Effects of Bread Fortification With Pomegranate Peel Powder on Inflammation Biomarkers, Oxidative Stress, and Mood Status in Patients With Type 2 Diabetes: A Randomized Placebo‐Controlled Trial

**DOI:** 10.1002/fsn3.70577

**Published:** 2025-08-13

**Authors:** Maryam Zare, Mohammad Javad Tarrahi, Omid Sadeghi, Mozhgan Karimifar, Sayed Amir Hossein Goli, Reza Amani

**Affiliations:** ^1^ Department of Clinical Nutrition, School of Nutrition and Food Science Isfahan University of Medical Sciences Isfahan Iran; ^2^ Epidemiology and Biostatistics Department, School of Public Health Isfahan University of Medical Sciences Isfahan Iran; ^3^ Nutrition and Food Security Research Center, Department of Community Nutrition, School of Nutrition and Food Science Isfahan University of Medical Sciences Isfahan Iran; ^4^ Isfahan Endocrine and Metabolism Research Center Isfahan University of Medical Sciences Isfahan Iran; ^5^ Department of Food Science and Technology, College of Agriculture Isfahan University of Technology Isfahan Iran

**Keywords:** bread, diabetes mellitus, inflammation, pomegranate

## Abstract

We hypothesized that bread fortified with pomegranate peel powder (PPP) would improve inflammation, oxidative stress, and mood in patients with type 2 diabetes mellitus (T2DM). To confirm this, its effect on the parameters above was evaluated. In total, 90 T2DM patients were randomized to receive either bread fortified with 3.5% PPP (*N* = 45) or PPP‐free bread for 12 weeks (*N* = 45). Dietary intake throughout the trial was assessed via food records. Laboratory parameters, including total antioxidant capacity (TAC), malondialdehyde (MDA), high‐sensitivity C‐reactive protein (hs‐CRP), and psychological disorders, including depression, anxiety, and stress, were assessed at the beginning and end of the study. 77 diabetic patients completed the trial (PPP group = 39 and control group = 38). Based on the compliance assessment, adherence to the interventions was high in the trial. We detected significant reductions in hs‐CRP levels (intervention group: change = −0.56 ± 1.29, *p* = 0.01; control group: change = −0.81 ± 1.16, *p* < 0.001) and depression scores (intervention group: change = −1.33 ± 3.66, *p* = 0.04; control group: change = −1.44 ± 2.83, *p* = 0.01). There was no significant difference between the groups. After adjusting for confounding factors, the important effect within the group disappeared. Other variables, including MDA, TAC, anxiety, and stress, did not significantly change in the two study groups. Whereas 12 weeks of consumption of PPP‐fortified bread failed to significantly affect oxidative stress, hs‐CRP levels, or mental state in T2DM patients, partial beneficial effects on inflammation and mood suggest the need for further research.

## Graphical abstract

This is a clinical trial study on the effect of bread with peel pomegranate on type 2 diabetic patients. According to the results, there was no significant improvement in stress oxidative, inflammation, and mental health in the participants.

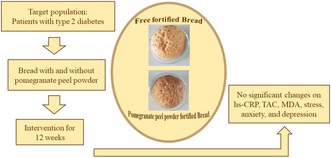

## 1 Introduction

Type 2 diabetes mellitus (T2DM) represents a growing global health crisis, with adults affected by this condition comprising 9.3% of the global population (Khan et al. 2020; Saeedi et al. 2019). In Iran, an estimated 9.2 million individuals are living with T2DM (Saeedi et al. 2019). In addition to foot ulcers, dental disease, and decreased resistance to infections, patients with T2DM usually experience several complications, which negatively affect their quality of life (Polikandrioti et al. 2020; G. Aktas 2023, 2024). Many chronic diseases are also more likely to develop in these patients, including cardiovascular disease (CVD), nephropathy, retinopathy, and liver disease (Martín‐Timón et al. 2014; Kosekli and Aktas 2025). The pathophysiology of T2DM and its associated complications is characterized by chronic systemic inflammation and oxidative stress (Balci et al. 2024; Basaran and Aktas 2025). Hyperglycemia drives the generation of reactive oxygen species (ROS), creating a cascade of inflammatory responses that perpetuate disease progression (Balci et al. 2024). This inflammatory burden extends beyond T2DM itself to related metabolic disorders including prediabetes, metabolic syndrome, hepatosteatosis, and obesity (Balci et al. 2024). Furthermore, the interplay between chronic inflammation, oxidative stress, dietary factors, and psychiatric comorbidities such as depression and anxiety creates a complex web of pathogenic contributors that influence glycemic control (Balci et al. 2024). Given this multifactorial etiology, effective diabetes management must extend beyond traditional glycemic control to address these underlying inflammatory and oxidative pathways. This comprehensive approach has led researchers to explore both pharmacological and non‐pharmacological interventions, with particular interest in dietary modifications, nutrient supplementation, and herbal medicine due to their favorable safety profiles (Cannon et al. 2018; Leite et al. 2020; Evans and Bahng 2014). Recent evidence suggests that polyphenol‐rich foods and supplements can significantly improve clinical outcomes in diabetic patients through their potent antioxidant and anti‐inflammatory properties (Raimundo et al. 2020). These bioactive compounds specifically target key modifiable risk factors including metabolic syndrome, dyslipidemia, obesity, and elevated inflammatory markers. Among polyphenol‐rich foods, pomegranate and its by‐products have garnered considerable attention as functional foods with therapeutic potential (Salama et al. 2021).

Pomegranate peel powder (PPP) represents a particularly promising intervention, as it contains concentrated bioactive compounds and dietary fiber while being low in phytic acid, making it suitable for food fortification applications (Sulieman et al. 2016). Preliminary clinical evidence demonstrates that pomegranate peel extract can significantly reduce high‐sensitivity C‐reactive protein (hs‐CRP) levels in diabetic patients, indicating its anti‐inflammatory potential (Haghighian et al. 2016).

However, research on the therapeutic effects of PPP incorporated into commonly consumed food items remains limited. Bread represents an ideal vehicle for functional food fortification given its widespread consumption globally (Nikooyeh et al. 2016). In the Iranian dietary context, this approach holds particular relevance, as over 60% of daily energy intake derives from carbohydrates, predominantly from bread consumption (Heidari et al. 2019). Despite promising preliminary findings with fortified foods, their adoption remains uncommon in Iran, presenting an opportunity for novel dietary interventions.

Therefore, this randomized clinical trial was designed to evaluate the effects of PPP‐fortified bread on inflammatory markers (hs‐CRP), oxidative stress parameters, and mental health indicators in patients with T2DM.

## 2 Methods and Materials

### 2.1 Study Population

A randomized, blind, parallel clinical trial was conducted in this study. Previously, details on participants, study design, and data collection were published (Zare et al. 2023). From May 2021 to January 2023, participants attended the outpatient clinic of the Endocrine and Metabolism Research Center, Isfahan University of Medical Sciences, Isfahan, Iran. The required sample size was calculated based on a type 1 error of 5%, a type 2 error of 20%, and a study power of 80%. We reached 40 participants per group. Finally, assuming a 10% dropout rate, 45 patients were considered for each group (Haghighian et al. 2016). The main inclusion criteria were: (1) Patients with T2DM within the last 5 years, (2) patients aged 40–60 years, (3) those with HbA1c levels between 5.7% and 8.0% over the past 3 months, and (4) patients with a body mass index (BMI) ≤ 30 kg/m^2^. Cirrhosis, hepatitis, pregnancy, lactation, insulin use, adherence to a special diet, and antioxidant supplements within the last 3 months were excluded. Patients who changed dosages or types of medications, those unwilling to continue the trial, and those reporting complications were excluded from the trial. Informed consent was obtained from all participants by the Helsinki Declaration. A protocol for the present trial was approved by Isfahan University's Ethical Committee (approval no. IR. MUI.RESEARCH.REC.1399.087) and registered at www.IRCT.ir (ID: IRCT20191209045672N1). In addition, our study was reported in line with the guidelines provided by the Consolidated Standards of Reporting Trials statement (CONSORT) (Bennett 2005).

### 2.2 Study Design

A total of 11,165 patients were evaluated, but only 520 diabetics met the inclusion criteria. A total of 122 patients agreed to participate in the study. In the run‐in period, 22 patients were excluded because they were unwilling to follow the trial. Several reasons contributed to their exclusion, including distance from their homes, COVID‐19 strikes, and uncontrolled blood sugar levels. Participants were included in the study if their fasting blood glucose was less than 150 mg/dL at the first visit (Wei et al. 2014). As a result of the run‐in period, 10 participants were excluded due to their high levels of HbA1c (glycated hemoglobin). After that, the remaining 90 patients were randomly divided into 2 equal groups using computer‐generated random numbers. Groups of patients were assigned to consume either PPP‐fortified or PPP‐free bread. The shape, size, and packaging of both types of bread were identical. Breads had the same appearance, due to their remarkable resemblance, it was difficult to differentiate between them. Patients received control bread during the run‐in period. Each patient received two sessions during the run‐in period. The adherence to bread intake and possible complications related to bread intake were assessed in each session. All patients (*n* = 90) were followed for 12 weeks after the run‐in period. During the trial, the investigator and patients communicated via text messaging, phone calls, and face‐to‐face meetings. During the study, they were also asked not to change their usual diet or physical activity. A fasting blood sample and mental health data were collected from each participant at baseline and the end of the trial. The adherence to the intervention was evaluated using dietary records. Moreover, compliance with bread intake was monitored through routine visits and weekly phone interviews. It was, however, not possible to identify biochemical indicators associated with bread consumption.

### 2.3 Dietary Intake and Physical Activity Assessment

At baseline and the end of the trial, three‐day physical activity records were used to assess physical activity. We also assessed usual dietary intake before and after the intervention via two 3‐day food records. The food and physical activity records were explained by a nutritionist. Nutritionists checked the records; registered nutritionists reviewed and verified the dietary intake records for accuracy and completeness. A food album was provided for them to record their intake accurately. A conversion of serving sizes to grams per day was carried out after the dietary records were collected. Using Nutritionist IV software, we calculated the average energy and nutrient intake for each 3‐day record (First Data Bank; Hearst Corp, San Bruno, CA, USA).

### 2.4 Pomegranate Peel Powder

Pomegranate fruits used in current research were harvested from Fars Province, Iran. The selected variety, “Rabab,” is particularly famous for its fruit size, shiny red arils, and well‐balanced sweet‐tart flavor. It is also one of the most commercially valuable pomegranate varieties cultivated in Iran. From a botanical point of view, the pomegranate ( *Punica granatum* L.) is a member of the family Lythraceae (Zarei et al. 2010; Zeynalova and Novruzov 2017). PPPs were separated, dried, and ground into powder. The PPP mixture was stored at −20°C until the bread was prepared. PPP‐rich bread was prepared by adding 3.5 g of PPP per 100 g of wheat flour. This dose was considered safe and acceptable based on findings from previous studies and results from our sensory panel, which affirmed its acceptability in terms of flavor, texture, and overall acceptability (Sayed‐Ahmed 2014). Bread preparation also involves sourdough, rye flour, salt, sugar, yeast, and water. In each 100‐g bread, there was 63 g plain flour, 14 g rye flour, 14 g whole wheat flour, 2 g gluten, 1.4 g yeast, 0.14 g brown sugar, 0.1 g improver, 0.8 g salt, 1.4 g sourdough. PPP was the only difference between the pomegranate bread and the control bread. A company in Isfahan, Iran, produces both types of bread.

### 2.5 MTT (3‐[4,5‐Dimethylthiazol‐2‐Yl]c‐2,5 Diphenyl Tetrazolium Bromide) Test

Based on the Badano JA method, pure PPP was measured by MTT against fibroblast L929 cells (Pasteur Institute, Tehran, Iran) (Badano et al. 2019). Briefly, 100 μL of cell suspension in RPMI‐1640 medium (Gibco, Thermo Fisher Scientific, USA) (1 × 10^4^ cells/well) was seeded into each well of a 96‐well microplate and then incubated for 24 h (37°C and humidified 5% CO_2_ air). After that, the old medium was removed, and 100 μL of different concentrations of PPP were added to each well and incubated for another 24, 48, and 72 h under similar conditions. Consequently, 10 μL of MTT solution (5 mg/mL PBS, Sigma‐Aldrich, USA) was added to each well of the plates in the dark and incubated at 37°C for 2–3 h. To dissolve the formed formazan crystals, the old media containing MTT was removed, and DMSO (Sigma‐Aldrich, USA) (100 μL) was gently added to each well. The optical density of each well was measured with an ELISA plate reader (Startfix‐2100, Awareness, USA) at 570 nm. DMSO (1%) and doxorubicin were used as negative and positive controls, respectively. The concentrations that inhibited half of the cell population (IC50) were obtained by modeling the percentage of cytotoxicity versus the concentration of PPP. The assay was performed in triplicate, and the results are reported as the means ± standard deviations (SDs). The cell viability (%) was determined using Equation (1).

(1) $$\begin{array}{c} \%\text{Cell viability}\\ =\;\frac{\text{Absorbance of treated cells}-\text{background absorbance}\left(b\right)\;}{\text{Absorbance of untreated cells}\left(c\right)-\text{background absorbance}\left(b\right)\;}\times 100 \end{array}$$

where b = blank and c = control.

### 2.6 Assessment of Variables

Using an interview‐based questionnaire, age, sex, education, medication, supplement usage, and history of diseases were collected. Standard techniques were used to measure anthropometric measurements, such as the height and weight of the subjects. M.Z. performed the measurements in the morning. Using a stadiometer, the height was measured to the nearest 0.1 cm without footwear (Seca, Hamburg, Germany). Using a Seca Beam Balance, weight was measured to the nearest 0.1 kg while wearing light clothing (Seca, Hamburg, Germany). The BMI was calculated using the following formula: weight (kg)/height (m^2^) (kg/m^2^). Laboratory parameters and psychological factors were collected at baseline and 3 months after intervention.

### 2.7 Laboratory Measurements

At the beginning and end of the intervention period, blood samples (10 mL) were taken after 12 h of overnight fasting. Then, the serum samples were separated and stored at −80°C until further analysis. Malondialdehyde (MDA), HbA1c, and hs‐CRP were measured as primary outcomes. A latex immunoturbidimetric assay was performed to measure hs‐CRP (aptec, Belgium). A standard kit was used to measure serum TAC using the cupric‐reducing antioxidant capacity method (Kiazist Life Sciences, Iran). Thiobarbituric acid reactive substances (TBARS) were used to estimate MDA (Kiazist Life Sciences, Iran). The HbA1c levels were measured using a BT1500 device through turbidimetric immunoassay (Biotecnica, Rome, Italy).

There was an intra‐assay CV of 10% and an inter‐assay CV of 8% for all kits.

### 2.8 Mental Health

Psychological outcomes including depression, anxiety, and stress were assessed using the Depression Anxiety Stress Scales‐21 (DASS‐21). Mood state was also evaluated at baseline and 12 weeks using the validated Persian version of the Depression, Anxiety, and Stress Scale‐21 (DASS‐21) self‐report measure. It consists of three subscales, each with seven items rated on a 4‐point Likert scale (0–3), representing the severity of symptoms in the previous week (Cannon et al. 2018; Maroufizadeh et al. 2014).

### 2.9 Statistical Analysis

A per‐protocol approach was used for statistical analyses. Per‐protocol analysis included data from those who completed the intervention. Skewness and the Kolmogorov–Smirnov test were used to assess the normality of continuous variables. In the current study, all outcome variables were normally distributed. An independent samples *t*‐test was used to determine the differences between the intervention and control groups regarding continuous variables. To determine the distribution of categorical variables between the 2 groups, the chi‐square test was used. A paired sample *t*‐test was used to compare within subjects in each group. Additionally, changes in outcome variables were compared between the two groups via the independent samples *t*‐test. To assess the effects of PPP‐fortified bread on inflammation and mental health, we used analysis of covariance (ANCOVA and MANCOVA) with age, sex, and weight as covariates.

A *p*‐value of less than 0.05 was considered significant. The statistical analysis was conducted using SPSS version 20 (SPSS Inc., Chicago, IL, USA).

## 3 Results

### 3.1 Cytotoxicity and IC50

**FIGURE 1:**
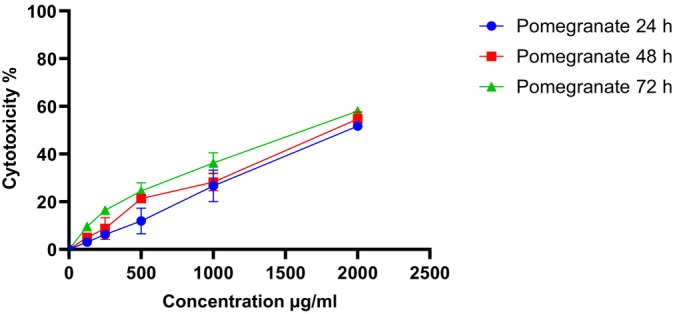
Cytotoxic effects of pomegranate peel powder on L929 fibroblasts.

**FIGURE 2:**
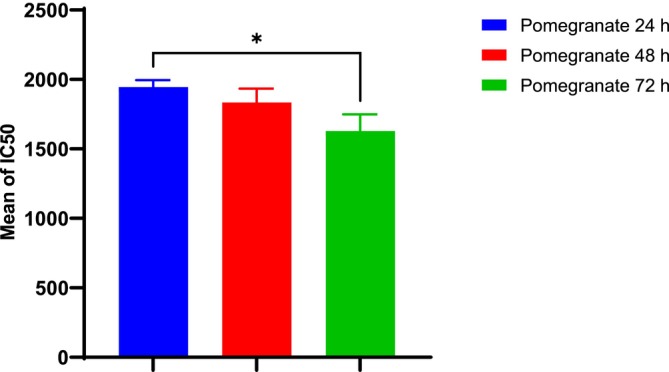
Comparison of the IC_50_ values of pomegranate compounds at 24, 48, and 72 h in fibroblast lines via Tukey's multiple comparisons test. *p ≤ 0.05.

**FIGURE 3:**
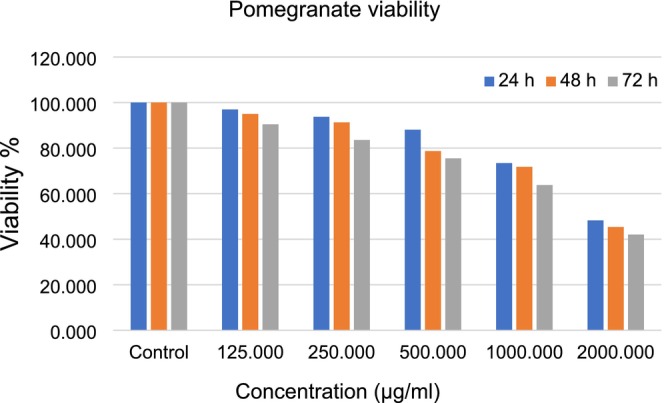
Cell viability after treatment with different concentrations of pomegranate peel powder.

The results of the cytotoxicity of the PPP against the fibroblast L929 cell line are presented in Figures 1, 2, 3. As shown in Figure 1, increasing the concentration of PPP from 125 to 1900 μg/mL increased the cytotoxicity against fibroblasts (Figure 1). There was no significant difference in the IC50 of PPP after 24 h and 48 h, confirming that the toxicity of PPP was not time‐dependent until 48 h. The cytotoxicity increased after 72 h, and there was a significant difference (*p* = 0.05) between 24 h and 72 h, which is explained by the release of antioxidant compounds from PPP (Figure 2). Moreover, the cell viability at different concentrations decreased with increasing concentrations from 125 to 1900 (μg/mL) (Figure 3).

### 3.2 Baseline Characteristics and Dietary Intake

**FIGURE 4:**
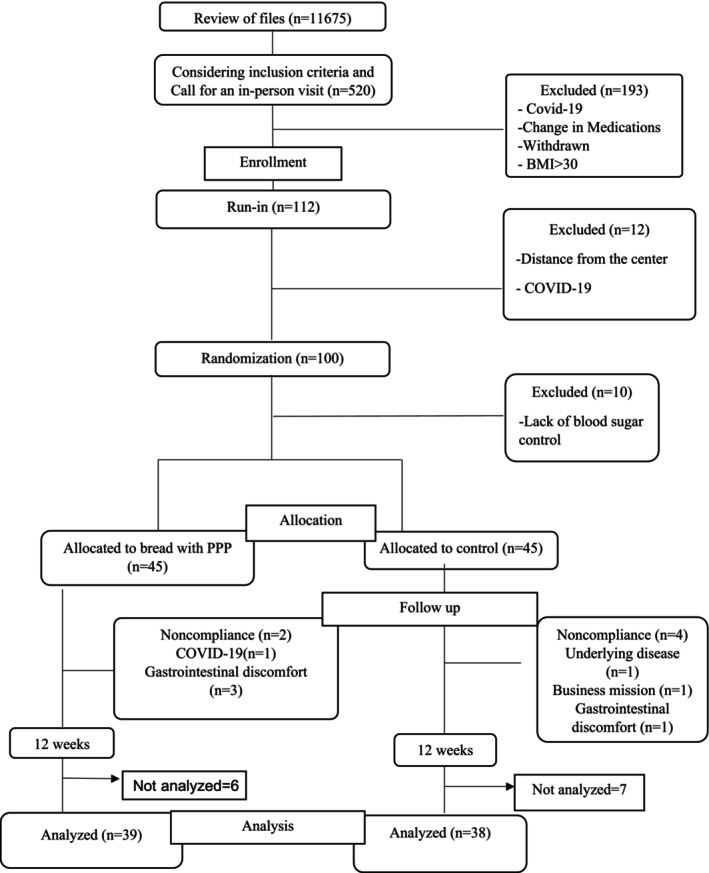
Study flow diagram based on the CONSORT statement.

َA total of 90 participants were enrolled in the study based on the inclusion criteria. During the study, 6 patients from the intervention group and 7 patients from the control group withdrew. The intervention period was completed by 77 subjects (39 in the intervention group and 38 in the control group). Figure 4 illustrates the flow diagram of this study. In Table 1, we present the baseline characteristics of the PPP‐fortified bread and control groups with T2DM. No significant differences were found between the two groups according to demographic variables, medical history, drug and supplement use, or anthropometric measurements. Furthermore, there were no significant differences between the groups in terms of physical activity. There were no significant differences in energy, macronutrients, and micronutrient intake between the two groups based on the 3‐day food records. Furthermore, no statistically significant differences were found between the groups regarding fiber, omega‐3 fatty acids, as well as vitamins and minerals with antioxidant properties (Table 2).

**TABLE 1:** Baseline characteristics of the study participants^a^.

Variable	Bread with PPP	Control	*p* ^b^
Sex			0.13
Female (%)	53.3 (24)	68.9 (31)	
Age	51.88 ± 5.10	55.02 ± 4.97	0.004
Body weight (kg)	72.51 ± 8.41	69.29 ± 9.67	0.09
HbA_1_C (%)	6.51 ± 0.96	6.49 ± 0.97	0.92
Physical activity (MET/h/day)	32.33 ± 4.48	32.55 ± 4.91	0.84
Education level *n* (%)			
Diploma and university degree	77.8 (35)	80 (36)	0.79
Medication use			
Anti‐diabetic; %(N)	88.9 (40)	77.8 (35)	0.16
Statins; %(N)	66.7 (30)	60 (27)	0.51
Anti‐depressants; %(N)	13.3 (6)	4.4 (2)	0.26

^a^ Values are presented as the means ± SDs and percentages for quantitative and qualitative variables, respectively.

^b^ Results from the chi‐square test for qualitative variables and the independent *t*‐test for quantitative variables.

**TABLE 2:** Dietary intake at baseline and after 12 weeks of intervention^a^.

Nutrients	Intervention (*n* = 45)	Control (*n* = 45)	*p* ^b^
Energy (kcal/day)			
Week 0	1998.98 ± 468.98	1979.65 ± 444.51	
Change	−61.17 ± 383.92	−116.98 ± 318.76	0.57
Carbohydrate (g/day)			
Week 0	300.16 ± 79.44	306.15 ± 78.60	
Change	−10.59 ± 63.03	−21.15 ± 49.56	0.50
Protein (g/day)			
Week 0	69.39 ± 19.51	70.82 ± 14.97	
Change	−2.11 ± 21.70	−3.28 ± 16.73	0.82
Fat (g/day)			
Week 0	62.26 ± 20.67	55.83 ± 19.48	
Change	−1.87 ± 21.50	−2.00 ± 21.67	0.98
Omega‐3 (g/day)			
Week 0	0.66 ± 0.34	0.66 ± 0.35	
Change	−0.05 ± 0.39	−0.10 ± 0.42	0.68
Fiber (g/day)			
Week 0	17.26 ± 5.30	17.45 ± 6.08	
Change	−0.11 ± 5.17	0.00 ± 4.57	0.93
Vitamin C (mg/d)			
Week 0	109.50 55.32	117.82 ± 65.58	
Change	9.86 ± 84.54	−1.08 ± 70.23	0.61
Vitamin E (mg/d)			
Week 0	20.97 ± 8.40	20.59 ± 11.66	
Change	−0.58 ± 11.53	−1.20 ± 13.78	0.85
Selenium (mcg/d)			
Week 0	0.08 ± 0.03	0.08 ± 0.03	
Change	0.00 ± 0.05	0.00 ± 0.04	0.60
Zinc (mg/d)			
Week 0	7.2 ± 2.3	6.98 ± 1.96	
Change	0.24 ± 2.93	0.11 ± 2.79	0.87
Vitamin A (mcg/d)			
Week 0	983.58 ± 784.04	798.69 ± 454.00	
Change	−58.20 ± 1169.33	−210.45 ± 920	0.60
Beta‐carotene (mcg/d)			
Week 0	397.35 ± 716.74	275.25 ± 311.68	
Change	82.19 ± 648.73	−61.23 ± 362.13	0.33

^a^ Values are reported as the mean ± SD.

^b^ Obtained from independent *t*‐tests.

### 3.3 The Effect of PPP‐Fortified Bread on Oxidative Status and Inflammatory Marker

Table 3 shows the effect of PPP‐fortified bread on hs‐CRP, TAC, and MDA serum concentrations and mental health. The hs‐CRP levels were 2.75 ± 1.56 at baseline and 2.19 ± 2.03 at the end of the trial in the intervention group and 2.45 ± 1.38 at baseline and 1.64 ± 1.19 at the end of the trial in the control group. The levels of hs‐CRP decreased in both groups, but the differences between the groups were not significant. The reduction in hs‐CRP levels indicated an effect size of 0.81 in the control group and 0.56 in the intervention group.

**TABLE 3:** Effects of treatment with PPP on inflammation, oxidative stress markers and mood scores^a^.

Variables	Intervention		*p* ^b^	Control		*p* ^b^	*p* ^c^	*p* ^d^
	Pre	Post		Pre	Post			
Hs‐CRP (mg/dL)	2.75 ± 1.56	2.19 ± 2.03	0.01	2.45 ± 1.38	1.64 ± 1.19	< 0.001		
Change	−0.56 ± 1.29			−0.81 ± 1.16			0.37	0.30
MDA (nmol/mL)	232.51 ± 104.87	217.67 ± 70.47	0.447	236.09 ± 76.84	213.24 ± 53.03	0.07		
Change	−14.84 ± 117.36			−22.85 ± 75.33			0.72	0.87
TAC (nmol/mL)	1467.92 ± 309.77	1513.87 ± 395.99	0.34	1606.35 ± 303.60	1608.16 ± 324.34	0.96		
Change	45.94 ± 298.39			1.81 ± 274.49			0.50	0.49
Depression	6.66 ± 4.60	5.33 ± 4.36	0.04	5.89 ± 4.32	4.44 ± 4.28	0.01		
Change	−1.33 ± 3.66			−1.44 ± 2.83			0.89	0.65
Anxiety	5.30 ± 4.11	6.03 ± 3.80	0.17	4.72 ± 3.89	5.62 ± 3.25	0.08		
Change	0.72 ± 3.00			0.89 ± 2.73			0.81	0.92
Stress	9.27 ± 6.09	8.15 ± 5.48	0.24	7.13 ± 5.24	6.86 ± 5.73	0.66		
Change	−1.12 ± 5.42			−0.27 ± 3.42			0.47	0.45

Abbreviations: hs‐CRP, high‐sensitivity C‐reactive protein; MDA, malondialdehyde; TAC, total antioxidant capacity.

^a^ Variables are expressed as the mean ± SE.

^b^ Calculated by paired‐samples *t*‐test.

^c^ Calculated via an independent *t*‐test.

^d^ Obtained from multivariable analysis of covariance (MANCOVA), adjusted for age, sex, and baseline weight.

Neither group showed significant differences in TAC and MDA.

### 3.4 The Effect of PPP‐Fortified Bread on Mental Health

The depression scores were lower (6.66 ± 4.60 at baseline vs. 5.33 ± 4.36 at the end of the trial, *p* = 0.04) in the intervention group and (5.89 ± 4.32 at baseline vs. 4.44 ± 4.28 at the end of the trial) in the control group. The reductions in the two groups were significant, although the changes between the two groups were not significant. The calculated effect sizes were approximately 0.30 in the intervention group and 0.34 in the control group, indicating small to moderate within‐group improvements over the course of the trial.

Neither group showed significant differences in stress scores. By controlling for age, sex, and weight, no differences were found.

### 3.5 Side Effects

Constipation and stomach discomfort were the only reported adverse events (3 patients in the intervention group and 1 patient in the control group).

## 4 Discussion

T2DM represents a growing global health challenge, emphasizing the critical need for effective disease management strategies. This randomized controlled trial was designed to evaluate the therapeutic potential of PPP‐fortified bread on oxidative stress biomarkers, inflammatory indicators, and mental health parameters in diabetic patients. While both intervention groups demonstrated improvements in hs‐CRP levels and depression scores following the intervention period, no statistically significant between‐group differences were observed for oxidative stress, inflammation, or mental health outcomes when comparing PPP‐fortified bread to control bread.

Diabetes mellitus constitutes one of the most formidable challenges in contemporary public health (Amiri 2016). Individuals with diabetes consistently exhibit elevated inflammatory biomarker profiles compared to their non‐diabetic counterparts. Furthermore, the established association between inflammatory biomarkers and endothelial dysfunction renders diabetic patients particularly vulnerable to cardiovascular diseases (Medina‐Leyte et al. 2021). However, the therapeutic efficacy of incorporating PPP into staple foods like bread remains incompletely understood.

At the initiation of our research, clinical investigations examining PPP were notably absent from the literature. Subsequently, following our protocol registration with IRCT, a comparable study with reduced sample size was published (Akkuş et al. 2023). Our findings revealed improvement in hs‐CRP levels across both intervention and control groups after the 12‐week study period, though this improvement did not reach statistical significance when compared between groups. Previous research has predominantly investigated inflammatory responses using PPP supplements or concentrated extracts rather than PPP‐fortified food products.

Haghighian et al. demonstrated in their clinical trial that 8‐week PPP extract supplementation effectively reduced inflammatory biomarkers. Corroborating these findings, Grabež et al. reported significant reductions in inflammatory markers following a 6‐week intervention in both type 2 diabetic patients and obese women with dyslipidemia (Haghighian et al. 2016; Grabež et al. 2022). Additionally, diabetic patients consuming 250 mL daily of pomegranate juice experienced a substantial 32% reduction in hs‐CRP levels (Sohrab et al. 2014) These findings align with our observations and are supported by Salama et al., who previously demonstrated that PPP supplementation in high‐fat diet rats significantly attenuated systemic inflammation and vascular histopathology, potentially explaining the observed clinical benefits (Salama et al. 2021).

Elevated oxidative stress plays a pivotal role in the progression from prediabetes to overt diabetes and represents a fundamental pathogenic mechanism underlying numerous complications, including atherosclerosis, vascular inflammation, and endothelial dysfunction (Balci et al. 2024). Consequently, early identification and intervention targeting oxidative stress elevation are essential for preventing disease progression (Balci et al. 2024). Our investigation revealed no significant alterations in serum MDA and TAC levels throughout the study period. Despite the higher polyphenol content in PPP‐fortified bread compared to control bread, this enhanced antioxidant profile did not translate to measurable changes in MDA or TAC parameters. We acknowledge that future investigations would benefit from incorporating additional oxidative stress markers, including specific antioxidant enzymes (superoxide dismutase, catalase, glutathione peroxidase) and protein oxidation markers (protein carbonyls). While our current approach provides meaningful preliminary evidence regarding the antioxidant effects of PPP‐fortified bread, we recognize the necessity for more comprehensive oxidative stress profiling in subsequent investigations.

These findings are consistent with Akkuş et al., who reported that 8‐week supplementation with PPP‐enriched bread did not significantly impact TAC levels (Akkuş et al. 2023). Conversely, multiple investigations have documented significant MDA level reductions following pomegranate peel extract supplementation across diverse populations, including hemodialysis patients (Jafari et al.), osteoarthritis patients, and capsule formulations (Haghighian et al.). Similarly, Bagheri et al. observed decreased MDA levels in rats administered PPP extract (Jafari et al. 2020; Mahdavi and Javadivala 2021; Haghighian et al. 2021). The precise mechanisms through which pomegranate peel reduces lipid peroxidation and oxidative stress remain incompletely elucidated. Proposed mechanisms include reactive oxygen species (ROS) scavenging, enhancement of antioxidant enzymes such as paraoxonase, and modulation of transcription factor signaling pathways including NF‐κB, PPAR‐γ, and Nrf2. Experimental studies demonstrate that both PPP pretreatment and post‐treatment protocols suppress ROS production while augmenting hepatic antioxidant defense mechanisms.

Regarding anti‐inflammatory properties, PPP contains bioactive compounds including punicalagin and ellagic acid that inhibit proinflammatory cytokines through suppression of the MAPK pathway and NF‐κB activation. As a polyphenol‐rich compound, PPP effectively inhibits oxidative stress and downregulates inflammatory signaling cascades (Banihani et al. 2013; Shiner et al. 2007; Mukherjee et al. 2013; Vitale et al. 2017; Aggarwal and Shishodia 2004; Rosenblat et al. 2006).

Our study revealed a declining trend in depression scores across both groups; however, this reduction failed to achieve statistical significance in between‐group comparisons. Although within‐group depression score reductions were statistically significant, these improvements may not indicate clinically meaningful changes, particularly given the absence of significant between‐group differences. While we observed a statistically significant reduction in depression scores (mean change: −1.33 ± 3.66, *p* = 0.04), the modest effect sizes suggest that larger studies are necessary to establish clinically relevant psychological benefits of this intervention.

Clinical significance of Depression, Anxiety and Stress Scale‐21 (DASS‐21) changes was evaluated according to the three‐distribution clinical significance model, which categorizes patients as recovered, recovering, improved, unchanged, or deteriorated based on reliable change indices and movement between normative ranges (normal, outpatient, inpatient) (Ronk et al. 2013). Based on this analysis, PPP‐fortified bread appears to have minimal impact on psychological well‐being. These findings contrast with Barghchi et al., who reported that PPP supplementation combined with a weight loss diet effectively reduced depression and stress levels (Chen et al. 2023; Barghchi et al. 2023).

PPP contains bioactive polyphenols, particularly ellagic acid and punicalagin, which may contribute to mood enhancement through neuroprotective effects, neuroinflammation reduction, and modulation of neurotransmitter systems involved in emotional regulation, including serotonin and dopamine pathways (Chen et al. 2023). These compounds potentially enhance brain health and alleviate inflammatory stress, thereby playing a significant role in improving psychological well‐being (Chen et al. 2023). The quality of life in T2DM patients may be substantially impacted by psychological symptoms including depression, anxiety, and stress (Martino et al. 2019). The observed improvements in both groups may be attributed to the whole‐grain bread and sourdough formulation used in PPP‐fortified bread preparation, rather than the PPP fortification itself. Previous studies have established associations between whole grain consumption and depression reduction among Iranian adults (Sadeghi et al. 2020). Several factors may have contributed to the absence of significant findings in our investigation. The interaction between bread nutrients and PPP polyphenols may have diminished the expected effects on hs‐CRP and oxidative stress parameters. Polyphenol absorption may be impaired by fiber and other nutrients present in bread. Individual variations in polyphenol absorption, influenced by gut microbiota composition and dietary context, could have altered the bioavailability and biological activity of PPP compounds (Cardona et al. 2013). Additionally, within Iranian dietary culture, bread is typically consumed alongside complementary foods such as cheese, nuts, legumes, and dairy products. These co‐ingested items, rich in fiber, vitamins, and minerals, may interact with polyphenols and influence their absorption or metabolic processing. Storage temperature variations among participants' household freezers, which may not consistently maintain −20°C, could have affected bread quality and bioactive compound stability. Our study population comprises individuals with well‐controlled blood glucose levels, which may partially explain the absence of statistically significant changes.

The lack of significant changes in oxidative stress biomarkers may be attributed to several interconnected factors. First, the 3.5% PPP concentration, while optimized for palatability and practical application, may have provided insufficient bioactive compounds to overcome the chronic oxidative stress characteristic of T2DM. Second, the 12‐week intervention period, though adequate for detecting inflammatory changes, may have been insufficient for significant alterations in oxidative stress parameters, which often require extended exposure periods to demonstrate meaningful clinical changes. Future studies should consider dose–response investigations to determine optimal PPP concentrations, extended intervention periods (≥ 6 months) to allow for cumulative antioxidant effects, and alternative delivery methods to enhance bioavailability. We believe these findings contribute valuable preliminary data while highlighting important considerations for future research design. This investigation possesses several notable strengths. To our knowledge, this represents the first randomized clinical trial evaluating the clinical effects of PPP‐fortified sourdough bread in patients with type 2 diabetes. The study was conducted in a homogeneous population using rigorous inclusion–exclusion criteria. Throughout the intervention period, dietary intake and physical activity were carefully monitored. The inclusion of both genders enhances the generalizability of findings to diverse populations.

However, several limitations warrant consideration when interpreting our findings. We were unable to employ ELISA methodology for comprehensive oxidative stress assessment. Compliance evaluation could not be measured using specific biomarkers; however, dietary records were utilized to assess intervention adherence. Given our focus on type 2 diabetes patients, the generalizability of results to type 1 diabetes patients should be approached with caution. The daily provision of 100 g of bread to all participants makes it impossible to exclude the effects of non‐fortified bread components. The absence of comprehensive nutritional factor data, particularly polyphenol intake from other sources, represents another limitation.

Extended investigations are necessary to elucidate the long‐term effects of PPP‐enriched bread on oxidative stress and inflammation. Future studies should prioritize individuals with poorly controlled blood glucose levels, as they may demonstrate more pronounced metabolic responses. Given inter‐individual differences in polyphenol absorption, future investigations should employ multiple assessment methods, including serum, urine, and fecal analysis. To further clarify the effects of PPP‐enriched foods, studies involving participants with poorer metabolic control, extended intervention durations, and improved compliance and absorption measures may provide clearer insights into their therapeutic benefits. PPP may demonstrate greater efficacy in alternative food matrices such as dairy products or beverages; increased doses and extended durations may more clearly demonstrate its advantages. Future studies should consider incorporating additional inflammatory markers such as the systemic immune‐inflammation index (SII) and albumin/CRP ratio, which may enhance assessment capabilities while reducing costs (Kosekli and Aktas 2025; Wang et al. 2023; Bilgin et al. 2021).

## 5 Conclusion

In conclusion, PPP‐fortified bread did not significantly affect hs‐CRP levels, oxidative stress parameters, or psychological indices among individuals with T2DM compared to control bread. The capacity of both PPP‐fortified and control bread to reduce hs‐CRP and depression levels within groups suggests potential clinical significance. No serious adverse effects were associated with the PPP doses administered in this study. Future investigations examining PPP fortification in alternative food matrices, such as dairy products, would be valuable. Additionally, 3.5 g of PPP per 100 g of bread was determined to be safe and did not result in significant toxicity in MTT assay evaluation (3.5 g/100 g of bread).

## Author Contributions

**Maryam Zare:** conceptualization, formal analysis, methodology, resources, software, supervision, validation, writing – original draft, writing – review and editing, investigation. **Mohammad Javad Tarrahi:** formal analysis, methodology, writing review, and editing. **Omid Sadeghi:** formal analysis, software, writing – original draft. **Mozhgan Karimifar:** formal analysis, writing review, and editing. **Sayed Amir Hossein Goli:** methodology, writing – review, and editing. **Reza Amani:** project administration, methodology, resources, software, supervision, validation, writing – original draft, writing – review, and editing, investigation.

## Conflicts of Interest

There are no conflicts of interest among the authors.

## Data Availability

The corresponding author will provide the datasets used and/or analyzed during the current study upon reasonable request.
